# Structural and functional correlations in a large animal model of bleomycin-induced pulmonary fibrosis

**DOI:** 10.1186/s12890-015-0071-6

**Published:** 2015-07-31

**Authors:** Louise Organ, Barbara Bacci, Emmanuel Koumoundouros, Garry Barcham, Marjorie Milne, Wayne Kimpton, Chrishan Samuel, Ken Snibson

**Affiliations:** Faculty of Veterinary and Agricultural Science, The University of Melbourne, Parkville, VIC Australia; Faculty of Veterinary and Agricultural Science, The University of Melbourne, Werribee, VIC Australia; Department of Electrical and Electronic Engineering, The University of Melbourne, Parkville, VIC Australia; Department of Pharmacology, Monash University, Clayton, VIC Australia

## Abstract

**Background:**

Idiopathic pulmonary fibrosis (IPF) is a severe and progressive respiratory disease with poor prognosis. Despite the positive outcomes from recent clinical trials, there is still no cure for this disease. Pre-clinical animal models are currently largely limited to small animals which have a number of shortcomings. We have previously shown that fibrosis is induced in isolated sheep lung segments 14 days after bleomycin treatment. This study aimed to determine whether bleomycin-induced fibrosis and associated functional changes persisted over a seven-week period.

**Methods:**

Two separate lung segments in nine sheep received two challenges two weeks apart of either, 3U bleomycin (BLM), or saline (control). Lung function in these segments was assessed by a wedged-bronchoscope procedure after bleomycin treatment. Lung tissue, and an ex vivo CT analysis were used to assess for the persistence of inflammation, fibrosis and collagen content in this model.

**Results:**

Fibrotic changes persisted up to seven weeks in bleomycin-treated isolated lung segments (Pathology scores: bleomycin12.27 ± 0.07 vs. saline 4.90 ± 1.18, n = 9, p = 0.0003). Localization of bleomycin-induced injury and increased tissue density was confirmed by CT analysis (mean densitometric CT value: bleomycin −698 ± 2.95 Hounsfield units vs. saline −898 ± 2.5 Hounsfield units, p = 0.02). Masson’s trichrome staining revealed increased connective tissue in bleomycin segments, compared to controls (% blue staining/total field area: 8.5 ± 0.8 vs. 2.1 ± 0.2 %, n = 9, p < 0.0001). bleomycin-treated segments were significantly less compliant from baseline at 7 weeks post treatment compared to control-treated segments (2.05 ± 0.88 vs. 4.97 ± 0.79 mL/cmH20, n = 9, p = 0.002). There was also a direct negative correlation between pathology scores and segmental compliance.

**Conclusions:**

We show that there is a correlation between fibrosis and correspondingly poor lung function which persist for up to seven weeks after bleomycin treatment in this large animal model of pulmonary fibrosis.

## Background

Idiopathic pulmonary fibrosis is a progressive and fatal disease due to the resulting functional impairment and respiratory failure caused by aberrant extracellular matrix remodelling in the lung parenchyma [1]. Whilst there are now two therapies available to slow the progression of the disease [2, 3], these therapies are unlikely to be effective in the entire spectrum of IPF patients and still do not address the fibrosis that results from aberrant wound healing and is a key contributor to organ failure.

The bleomycin model has become the most widely used and characterised animal model of pulmonary fibrosis to investigate IPF and typically involves an intratracheal administration of a single dose of bleomycin to induce fibrosis in the lung parenchyma [4]. Whilst bleomycin is considered the most effective method to induce reproducible and consistent experimental fibrosis, there is still concern regarding the temporal aspects and translation ability of this model [4]. The bleomycin model has two distinct phases: the inflammatory stage occurring within the first two weeks post injury which then subsides to the fibrotic phase [5, 6], which appears to have an active period followed by a late fibrosis phase [7].

The longevity of fibrosis in the rodent models is not well defined, with different durations reported between various studies. Resolution of fibrosis is one of the most commonly stated limitations of bleomycin models; based on reports showing that fibrosis is short-lived and usually resolves within six weeks post-injury [8, 9]. However, there are several studies that challenge this concept, with evidence that fibrosis is maintained over a longer period of time following either a single dose or multiple doses of bleomycin [7, 10–12]. For appropriate testing of anti-fibrotics therapies, intervention should ideally occur within the fibrotic phase of the injury response. Importantly, the fibrotic phase in the animal model utilised for the trial should be sustained for a lengthy period to determine the true drug efficacy, without being confounded by a natural resolution of the induced fibrosis. The uncertainty about the duration and persistence of fibrosis in some bleomcyin rodent models has led some researchers to cast doubt over the appropriateness of using this model to trial novel therapies, one of the primary uses of the model [9]. For this reason, it is critical to therefore determine whether a measurable fibrotic response is maintained for a sufficient period of time to trial novel therapies during the fibrotic phase in any animal model being considered for such experiments.

We have previously characterized a sheep model of pulmonary fibrosis using a two hit segmental infusion of bleomycin approach [13]. This model enables fibrosis to be produced within isolated lung segments, thus providing internal controls and the ability to control the severity of the fibrotic response induced without the risk of mortality or compromising the animals’ overall respiratory health, a common problem associated with traditional bleomycin models [11, 12]. Importantly, the method used in this model allows for reliable lung function data, such as lung compliance, to be sequentially collected in the same animals throughout the study. As the previous study examined the acute early phase of fibrosis, up to 2 weeks post-bleomycin, the purpose of the current study was to characterize later stages of segmental pulmonary fibrosis in the sheep model. We sought to determine what structural and functional changes persisted 7 weeks beyond the acute injury stage [13]. This time-frame was chosen based on the premise that if bleomycin-induced fibrosis was resolving in the sheep model, the improvement in lung pathology would be able to be identified at 6 weeks as reported for fibrosis resolution in some murine models. The overall goal of the study was to determine whether lung function deteriorated and fibrosis persisted in this model such that in future studies the model may be useful for testing clinically relevant anti-fibrotic agents during established and sustained fibrosis.

## Methods

### Experimental animals

Nine female merino sheep aged between 9 months and 1 year were utilised in the study. Animals were housed indoors and received anthelminthic to treat for any existing parasitic disease. The sheep were judged to be free from significant pulmonary disease on the basis of clinical examination before the commencement of experiments and on inspection of gross pathology at autopsy. The Animal Experimentation Ethics Committee of the University of Melbourne, which adheres to the Australian Code of Practice for the care and use of laboratory animals for scientific purposes, approved all experimental procedures outlined below.

### Bleomycin administration and treatment protocols

**Fig. 1:**
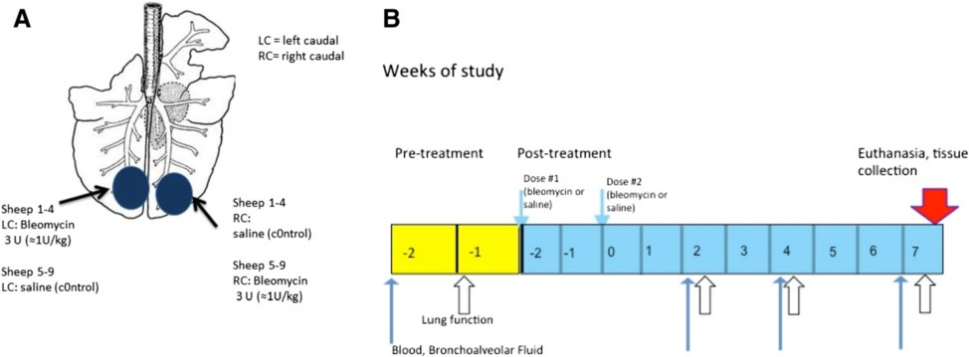
Challenge and sampling regime. Schematic diagram depicting the bleomycin and saline treatments administered to individual lung segments (**a**) two spatially distinct lung segments, which received the different treatments. The left caudal (LC) segment in sheep 1–4 received 3U bleomycin per segment, and the right medial (RC) segment received sterile saline as a control. The treatment and control segments were reversed in sheep 5–9 (**b**) Diagram indicating the time line for treatment administration, lung function and sample collection

Fibrosis was induced within specified regions of the lung of all treated animals, as indicated in Fig. 1a, using pharmaceutical grade bleomycin sulphate (Hospira, Melbourne, Australia). Bleomycin sulphate was made up at a concentration of 0.6 U bleomycin/mL saline as previously described [13]. Bleomycin solution was administered via the biopsy port of a fibre-optic bronchoscope to the appropriate lung segments as a 5 ml bolus (total dose of 3U/segment Fig. 1a). The bleomycin and saline control treatments were randomised between the most caudal segments of the left and right caudal lobes in different sheep. In particular, for sheep 1–4, bleomycin was infused into the left caudal lobes, while the right caudal lobes acted as internal controls and were with infused with 5 mL of sterile saline solution. These treatments were reversed between the lobes for sheep 5–9. Bleomycin and saline were first administered at week −2 and repeated two weeks later at week 0. (Fig. 1b). Sheep were then euthanized by an intravenous overdose of barbiturate (Lethabarb, Veterinary Clinic, Werribee, Australia) at week 7 as outlined in Fig. 1b; for tissue collection and analysis

### Necropsy and tissue sampling

Following euthanasia, the lungs were removed and targeted lung segments identified and carefully dissected free from surrounding tissue. Individual segments were then inflated with a 1:1 mixture of OCT and sterile PBS solution. This inflation procedure maintains lung segment tissues in an inflated state prior to either fixation in formalin, or freezing for cryo-sectioning. This allows unfixed tissues to undergo other processing procedures, as is standard procedure in our laboratory [13–15]. Several 2 mm thick transverse slices were taken and processed as follows: an area of lung slice derived from the most caudal region of each treated segment was fixed in 10 % neutral-buffered formalin and processed in paraffin for histopathology assessment. Remaining lung slices were embedded in OCT and frozen in cryo-moulds on aluminium boats floating on liquid nitrogen. These were kept at −80 °C for cryo-sectioning and immmunohistology.

### Analysis of segmental lung function

Segmental compliance (Cseg) was assessed using pressure responses to flow in the different segments as indicated Fig. 1a and b in awake, consciously breathing sheep using a wedged-bronchoscope technique as previously described [13] Briefly, a custom-built Segmental Lung Airway Monitoring (SLAM) System was used to monitor the segmental flow and pressure. After first determining the bronchoscope resistance to the set flow, the bronchoscope was wedged into an airway in the lung segment of interest and a constant flow (6 mL/s) of 5 % CO_2_ in AIR was passed through the working channel of the bronchoscope. Segmental lung compliance was calculated as previously described [13]. Briefly, after the bronchoscope was wedged into the specific region of the lung, pressure was allowed to reach a steady state. After approximately 5 s at steady state, airflow was interrupted turning off the airflow supply. Segmental compliance is then calculated from the pressure-flow decay curve generated from this procedure, as previously described [13, 16, 17]. The process was repeated 3 times for each segment and expressed as an average value for Cseg The pressure was recorded by a PM-1000 Transducer Amplifiers (CWE Inc., Admore, USA) and flow was recorded using a mass flow meter (824-S, SIERRA INSTRUMENTS, Monterey, USA). Data was acquired with Data Acquisition Card (PCI-6233; National Instruments Corp., Austin, USA) and was analysed with the SLAM system (Latitude E6520, Dell Computer Corporation, Texas USA and LabVIEW, National Instruments Corp., Austin, USA). All resistance measurements were corrected for the resistance of flow through the working channel of the bronchoscope.

### Histology

Paraffin-embedded tissue sections (5 μm) were stained with haematoxylin and eosin Y (H&E) for general histology and the assessment of pathological changes and Masson’s trichrome staining was used to identify changes to collagen content within the lung parenchyma as previously described [18]. Fibrotic lung injury was assessed morphologically by semi-quantitative and quantitative parameters as follows:

#### Semi-quantitative Morphological Index (SMI)

Histopathology of lung parenchyma was assessed by an experienced pathologist (BB) blinded to the treatment groups using a semi-quantitative scoring system, as previously described [13]. Briefly, the criteria used gives score indices separately for both inflammation and fibrosis pathology, and these score indices added together give an ‘overall pathology score’.

#### Quantitative Image Analysis (QIA)

- *Fibrosis fraction:* The degree of fibrosis, or collagen content, was quantified to give an indication of changes for overall collagen content within the parenchymal tissue using a previously described method [6]. Briefly, Masson’s trichrome stained slides were scanned into a digital format using Mirax slide scanner (Carl Zeiss Micro-Imaging, Jena, Germany). Ten consecutive, non-overlapping fields were selected for analysis, which lacked obvious airways and/or blood vessels. Each field was analysed using Image Pro plus (Version 6.3 for Windows, Media Cybernetics, Bethseda, Maryland, USA), using the ‘Colour selector’ tool to measure the area blue stained tissue (collagen) within the each field. The fraction of blue stained collagen areas for each of the ten fields was averaged for each slide. The area of the fraction of fibrosis is expressed as a percentage of the total field area.
- *Morphometrics:* Paraffin-embedded sections of lung tissue were stained with H&E for morphometric assessment. Digital images of lung parenchyma from control- and bleomycin-treated lung segments were imported into Image Pro Plus software for analysis. Measurements were made by superimposing custom-designed test grids over the lung parenchyma, which were generated using Image Pro. Tissue and airspace fractions were determined within parenchymal tissue by point-counting methods, as previously described [19, 20]. Analyses were performed from a total of 15 fields at a final magnification of x200.

### Ex-vivo CT analysis

CT analysis was performed on one set of lungs ex-vivo to localise the fibrosis and density changes in the lung. Selection was based on lung function results, with the sheep showing the most prominent loss in lung compliance chosen for the analysis. Following euthanasia, the lungs were carefully dissected out without damaging the parietal pleura and placed immediately on ice. The trachea was secured in an upright position to prevent any fluids filling the lungs.

CT scans were acquired using a 16 slice multirow detector CT scanner (Siemens Emotion 16, Siemens Erlangen, Germany). The lungs were placed in a water bath to allow for full inflation of the lungs and prevent artificial atelectasis from the pressure of the table. A 3 L syringe was tightly secured in the tracheal lumen and used to inflate the lungs throughout the scan. The scanner configuration was 16x0.6 mm; giving an effective slice of 0.75 mm, with a complete scan time of 97.75 s, at a pitch of 0.4, and rotation time of 1 s. The reference mAS was 70 and effective mAS 65. Two reconstructions of the acquired dataset were made: lung window with edge enhancement filter, soft tissue window with smoothing filter.

To analyse the tissue density, the reconstructed images were viewed using OsiriX Imaging software (Pixmeo, Switzerland, (19), which converted voxels to Hounsfield Units (HU) selecting air as −1000 and water as 0. The reconstructed image was loaded in the segmentation editor and the box selector tool was utilized to select the region of interest for saline and bleomycin-treated areas of lung. For the CT analysis, measurement of the tissue density was performed on 10 representative slices of the targeted regions to give a mean tissue density for each differentially treated lung segment.

### Collagen concentration

The hydroxyproline assay was used to extrapolate the collagen content and concentration of each segment, as described previously [21, 22]. Briefly, frozen lung tissues from each segment were lyophilized to dry weight, hydrolyzed in 6 M HCl, and assessed for hydroxyproline content by measuring the absorbance of reconstituted samples (in 0.1 M HCl) at 558 nm using a Beckman DU-64 spectrophotometer (Beckman Coulter Inc, Brea, CA). Hydroxyproline content was determined from a standard curve of trans-L-hydroxy-L-proline (Sigma-Aldrich). Collagen content was extrapolated by multiplying the hydroxyproline measurements by 6.94 (based on hydroxyproline representing ~14.4 % of the amino acid composition of collagen [23] in most tissues) and then expressed as a proportion of the dry weight tissue to yield collagen concentration (which was expressed as a percentage).

### Immunohistochemistry

Immunohistochemistry was performed on frozen tissue sections as previously described [14, 15]. Sections were fixed with 100 % cold ethanol for 10 min and were simultaneously blocked for endogenous peroxidase with 3 % H_2_O_2_ (Univar, Knoxville, Vic, Australia). Sections were then pre-blocked using blocking solution for 30 min (1 % bovine serum albumin, 5 % normal sheep serum in PBS). After blocking, sections were incubated with the primary antibodies (See Table 1). After washing, sections were incubated for with appropriate secondary antibodies (See Table 1) for 1 h. Sections were then washed and a peroxidase-based detection system was used for visualization. Specificity was determined by omission of the primary antibody on secondary controls, and biologically irrelevant isotype controls.

**Table 1:** Summary of antibodies used for immunohistochemistry

Primary Antibody	Supplier	Dilution	Incubation	Secondary Antibody
Mouse anti-αSMA	Sigma-Aldrich,	1:800	2 hrs	Rabbit anti-mouse Ig/HRP
Mouse anti-CD4	AbD Serotech, Raleigh, USA	neat	2 hrs	Rabbit anti-mouse Ig/HRP
Mouse anti-CD8	AbD Serotech, Raleigh, USA	neat	2 hrs	Rabbit anti-mouse Ig/HRP

### Lung parenchyma cell counts

Individual tissue sections immunohistochemically stained with one of CD4, CD8 (see above) were assessed for the number of positive cells in the parenchymal regions of the lung. Regions of intact lung parenchyma were visualized at 400x magnification using a microscope with graticule attachment. All positive cells within the boundaries of the graticule were counted and field of view was repositioned to a new area as necessary to obtain a count of at least 100 positive cells, recording both the number of fields and the total number of cells per sheep. The area of the graticule at 400x magnification was determined to be 0.078 mm^2^, this was used to calculate the cell density (cells/area; data is presented as cells/mm^2^).

### Collection of bronchoalveolar lavage

Bronchoalveolar (BAL) fluid was collected from all sheep 14 days prior to bleomycin administration from the respective lung segments and then again at 2 weeks, 4 weeks and 6 weeks (shown in Fig. 1b), as previously described [18]. BAL samples were collected at a minimum of 3 days either prior to, or after, lung function, with the final BAL samples taken the week prior to final lung function testing. The reason for the interval between lung function testing and BAL sampling is that if these two procedures are scheduled too close together, the first performed procedure influences parameters of the second performed procedure, leading to spurious data. To collect BAL cells/fluid, a flexible fibre-optic bronchoscope was advanced into the selected lung segments and a lavage was collected by instillation and withdrawal of approximately 10 mL aliquots of PBS solution. The samples were placed immediately on ice. The cells were separated from the supernatant by centrifuging the lavage fluid for 7 min at 1000 rpm to remove cells. Supernatant was stored at −80 °C until use in ELISA.

### TGF-β1 ELISA

Total TGF-β1 was assessed in BAL fluid by ELISA using commercially available kit, according to kit instructions (Quantikine; R&D Systems, Minneapolis, MN). Neat BAL samples were prepared for the assay following protocol for low protein samples, as indicated by the kit instruction. Results were normalized for protein concentration using a TGF- β1 standard and control (included in the kit).

### Statistical analysis

Statistical analysis was performed using GraphPad Prism for Windows Version 6.0 (GraphPad Software Inc, La Jolla, USA. Each parameter was assessed for Gaussian distribution both visually and using the D’Agostino & Pearson omnibus normality test. Comparisons between individual lobes within the same sheep and comparisons of pre- and post- treatment were performed using paired analyses. For normally distributed data, paired two-tailed *t* test were used. Wilcoxon matched-pairs signed rank test was used for data that did not meet assumptions required for parametric testing. To assess for associations between two measured parameters, Correlations were assessed by the Spearman correlation coefficient (rs) A P value of <0.05 was taken as significant. Values are reported as mean ± SEM, unless stated.

## Results

### Isolation of fibrosis to bleomycin-treated lung region

**Fig. 2:**
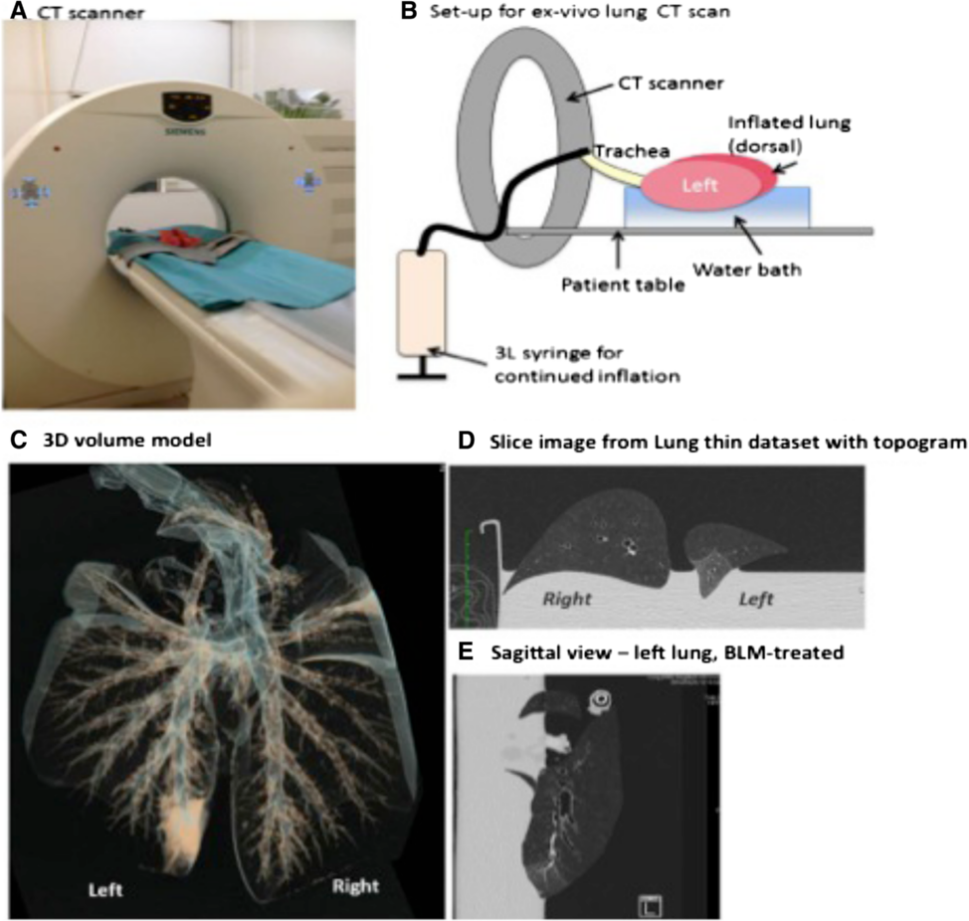
CT-analysis reveals isolation of fibrosis to specifically treated lung region. CT analysis was performed on one set of lungs ex-vivo to localise the fibrosis and density changes in the lung (**a**) Siemens Emotion 16 was used (configuration 16x0.6 mm, effective slice. Two reconstructions of the acquired dataset were made: Lung window with edge enhancement filter, Soft tissue window with smoothing filter. (**b**) Set-up of ex-vivo lung scans: Lungs were floated in a water bath and trachea was secured to a 3 L syringe to inflate the lungs throughout scan. Representative images obtained from CT scan highlighting the isolation of fibrosis to the bleomycin-treated (left caudal) lobe. Images show (**c**) Volume reformats (Blue 3D) model of the lungs. Areas of air show as hollow surrounded by the blue. Areas of increased density show up as the yellow colour. (**d**) Slice-image from reconstructed “Lung Thin” dataset, (**e**) Slice image from reconstructed sagittal reformats

To confirm that the fibrosis induced by the segmental administration of bleomycin was localized to the specifically treated lobes, an ex-vivo CT scan was performed on lungs removed at autopsy (Fig. 2). This scan confirmed that the bleomycin injury was confined to a small region of the lobe following administration using the bronchoscope, while the remainder of the lung has a normal radiological appearance (Fig. 2c-e). Measurements of the tissue density showed that the bleomycin-treated segment had significantly increased mean Hounsfield units compared with saline treated regions, which exhibited tissue density of a healthy lung (mean densitometry CT value: bleomycin −698 ± 2.95 Hounsfield units vs. saline −898 ± 2.5 Hounsfield units, p = 0.02, n = 1)

### Decreased lung compliance persists in bleomycin-treated segments

**Fig. 3:**
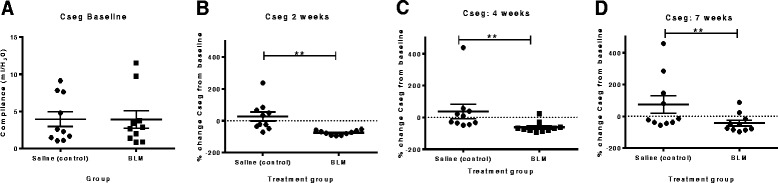
Decreased lung compliance persists in bleomycin-treated segments. Graphs show changes to segmental compliance values over time in the different lung segments (Cseg), as percentage change from pre-treatment values. **a** baseline compliance values, **b** Cseg at 2 weeks, **c** Cseg at 4 weeks and **d** Cseg at 7 weeks. Significance was determined using a matched-pairs *t*-test (Wilcoxin for non-normal data), ** p <0.01

Temporal changes in lung function in the individually treated segments were assessed in all sheep by measuring lung function before bleomycin or saline treatment, and again at 2, 4 and 7 weeks post –treatment. Our results showed that there was a significant decrease in the percentage change in compliance from baseline values observed at 2 weeks in segments treated with bleomycin compared to saline control segments (−75.9 ± 4.6 vs 26.5± 28.7 %, p=0.002, n=9, Fig. 3b). Lower lung compliance values persisted in the bleomycin treated segments throughout the remainder of the time-points assessed, as indicated by the significant difference between the bleomycin and saline-treated segments at both 4 and 7 weeks post bleomycin-treatment (4 weeks: −61.9 ± 10.73 vs 37.7 ±46.1 %, p=0.009, n=9, Fig. 3c and 7 weeks: − 42.5 ± 18.5 vs 75.2 54.8 %, p=0.002, n=9, Fig. 3d).

### Fibrotic remodelling changes present in bleomycin-treated segments up 7 weeks post-injury

**Fig. 4:**
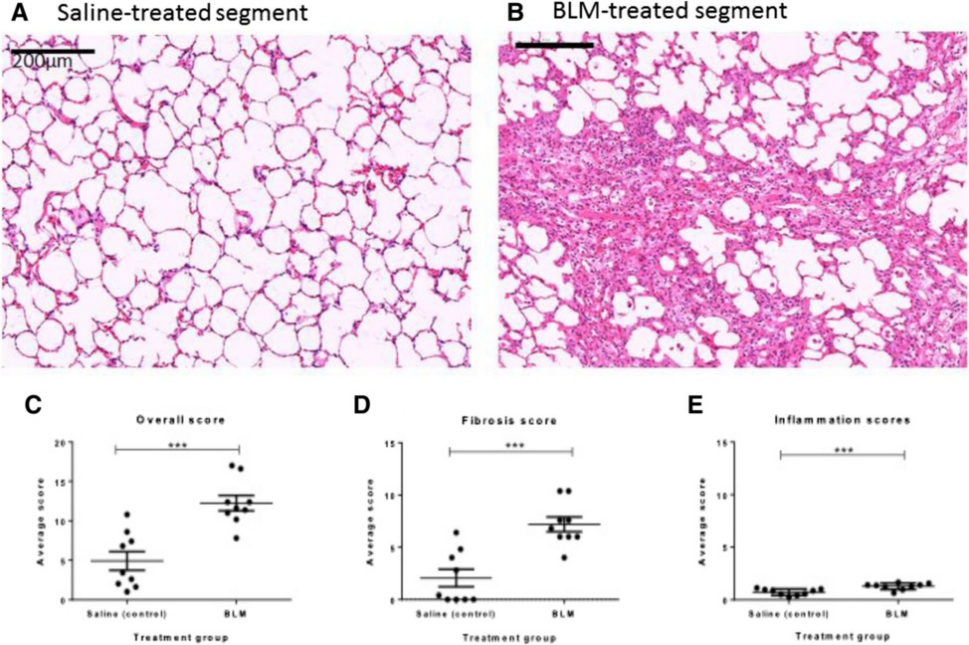
Fibrotic remodelling changes present in bleomycin-treated segments up 7 weeks post-injury. Panels depict histology of H&E stained sections of lungs segments taken at autopsy. **a** saline-treated segment. **b** bleomycin-treated segment, patchy areas of inflammation and fibrosis. Semi-quantitative scoring, shown is graphs (**c-e**) of the pathology revealed increased fibrosis and inflammation in bleomycin-treated segments at 7 weeks post-treatment, compared to saline treated controls (Graphs **c-e**.). Significance was determined using paired *T*-test (Wilcoxin for non-normal data) between treatment groups and unpaired T-tests between time points,***p<0.001

Histological examination was performed on both saline treated and bleomycin-treated lobes taken at autopsy and was quantified using a scoring system outlined in methods. The saline-treated segments all had normal architecture (Fig. 4a), with no prominent inflammation or fibrosis in the alveoli space or walls. There were no major pathological findings within the lung parenchyma, in line with typical healthy lung tissue (Fig. 4c, d, e).

**Fig. 5:**
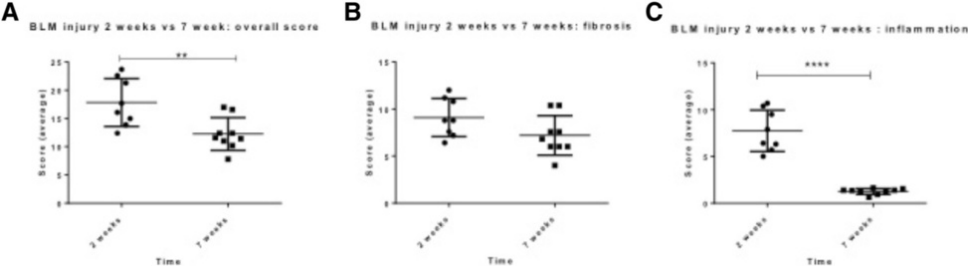
Reduction in inflammation over time. A comparison of injury scores at 7 weeks in this study to lung tissue taken 2 weeks post-injury from our previous study [13] showed that overall pathology was higher at 2 weeks than 7 weeks (Graph **a**), but the degree of fibrosis was similar between the two time-points (Graph **b**), whist the degree of inflammation was decreased at 7 weeks post-injury from inflammation observed at 2 weeks (Graph **c**). Significance was determined using unpaired T-tests between time points and is denoted, **p<0.01, ****p<0.0001

In bleomycin-treated lobes, extensive structural changes were observed in the lungs 7 weeks after the final bleomycin administration (Fig. 4b). Fibrosis was present in both alveolar wall and space, which had a multifocal appearance (Fig. 4b). Quantification of the injury showed that the overall injury score and fibrosis scores were significantly higher than that of saline treated controls (Overall Pathology scores: 12.27 ± 0.07 vs. 4.90 ± 1.18, p= 0.0003, n=9 Fig. 4c and Fibrosis scores: 7.2 ± 0.7 vs 2.0 ± 0.8, p=0.004, n=9, Fig. 4d). There was still some cellular infiltrate observed within the damaged regions of the lung, with inflammation scores were significantly elevated in comparison to saline treated lobes (1.3 ± 0.1 vs. 0.7 ± 0.1, p=0.0002, n=9, Fig. 4e). However, a comparison of the scores from the current study, at 7 weeks post-bleomycin are compared to our previous study [13], looking at injury 2 weeks post-bleomycin, indicates that the inflammatory scores at 7 weeks were significantly lower than those observed at the 2 week time-point (1.3 ± 0.1 vs. 7.7 ± 0.8, p<0.0001, n=8-9, Fig. 5c). There was a significant difference in the overall score between the two time points, which included the inflammatory score component (2 weeks vs. 7 weeks: 17.8 ± 1.5 vs 12.3 ± 0.9 p=0.009, n=8-9, Fig. 5a). Importantly, despite the reduced inflammation in the tissue at the seven-week post-injury time-point compared to the two-week time-point, there was no difference in fibrosis scores between these two time-points (2 weeks vs 7 weeks: 9.1 ± 0.7 vs 7.2 ± 0.7, p=0.08, n=8-9 Fig. 5b).

**Fig. 6:**
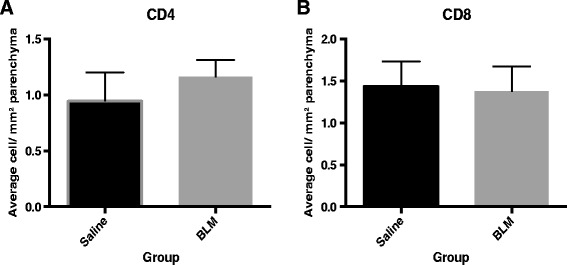
T cell subpopulations in the parenchyma in the different lobes at 7 weeks post injury were assessed on histological sections with antibodies raised against the respective antigens using a horseradish peroxidase based detection system. Counts expressed as cells per mm2 of parenchyma . The number of (**a**) CD4+ and (**b**) CD8+ Cytotoxic T cells helper cells in the bleomycin-treated lobes was not significantly different from the saline–treated lobes

Immunohistochemistry for CD4 and CD8 inflammatory cells revealed there was no significance difference in the number of either of these cell types between the saline control and bleomycin treated segments at seven weeks after bleomycin injury (CD4: 12.1 ± 3.3 vs. 14.9 ± 1.9 cells/mm^2,^ p=0.4, n=9, Fig. 6a; and CD8: 15.4 ± 4.4 vs. 14.7 ± 4.3 cells/mm^2^, p=0.9, n=9, Fig. 6b).

**Fig. 7:**
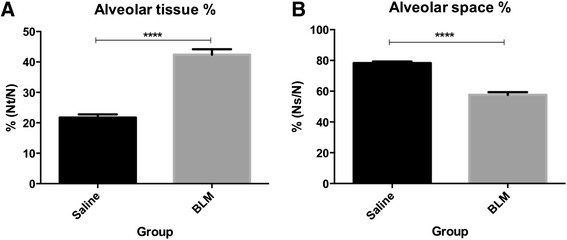
Alveolar airspace is significantly reduced in response to bleomycin (**b**). Changes to alveolar tissue percentage, showing increased tissue volume (**a**) and airspace percentage, showing decreased airspace volume (**b**), were assessed in H&E stained lung segments taken at autopsy. Measurements were taken from three differentially treated segments in each sheep using point-counting methods. Fifteen fields were assessed in each segment to obtain an average value. Values are shown as mean ±SEM for each segment for all animals, n=9. Significance was determined using a matched- ANOVA ****, p<0.0001

The percentages of tissue space and airspace were measured in the segments. Compared to the saline-treated controls, the mean percentage of tissue space was significantly higher in the bleomycin-treated segments (21.7 ±1.04 vs. 42.4 ± 1.8 %, p<0.0001, n=9, Fig. 7a). Correspondingly, the airspace percentage was significantly reduced in the bleomycin-treated segments, compared to saline-treated control segments (57.6 ±1.8 vs. 78.3 ±1.0 %, p<0.0001, n=9, Fig. 7b).

### Elevated collagen and connective tissue levels seven weeks post- injury

**Fig. 8:**
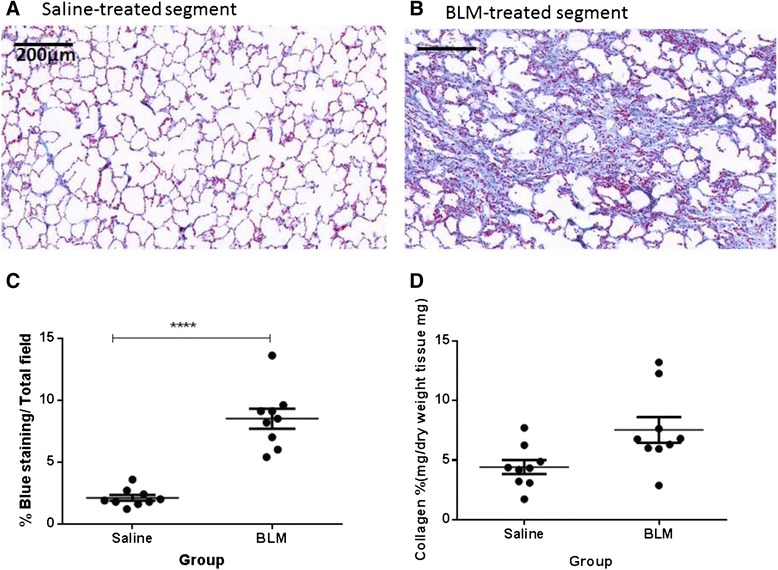
Increased connective tissue levels seven weeks post-injury, as assessed with Masson’s trichrome staining and hydroxproline assay for collagen. Representative images of Masson’s trichrome stained sections are shown for a saline-treated lung segment (**a**), and a bleomycin-treated segment lung segment (**b**). The fibrotic fraction of each treatment (area of blue staining area/total field area) is shown in (**c**). Results show mean ± SEM, n=8. The panel (**d**) shows collagen content as determined using hydroxyproline assay. Significance was determined using paired *T*-test ****p<0.0001

Masson’s trichrome staining and hydroxyproline assessments were used to determine the presence of connective tissue and collagen changes in the lungs seven weeks post bleomycin injury. An assessment of Masson’s trichome staining revealed the presence of extensive connective tissue in the remodelled regions of the bleomycin-treated lobes (Fig. 8b), compared to the minimal staining observed in saline-treated control (Fig. 8a), confirmed by quantification of the positive staining area in parenchymal tissue (8.5 ± 0.8 vs 2.1 ± 0.2 %, p<0.0001, n=9, Fig. 8c). The hydroxproline assay showed a measurable increase in lung collagen concentration levels in most bleomycin-treated lobes compared to their saline treated counterparts, but this did not reach statistical significance given the variation in data (7.5 ± 1.1 vs 4.4 ±0.5 %, p=0.06, n=9, Fig. 8d).

### Histopathology correlates to lung function changes

**Fig. 9:**
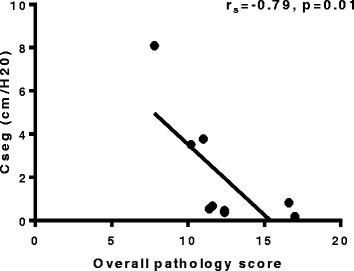
Histopathology correlates to lung function changes. Graph shows relationship between overall pathology score and lung compliance in bleomycin-treated segments was assessed (n=9). The measured compliance value (cm/H_2_0) at 7 weeks was correlated to the overall pathology score measured for the each corresponding segment

The histopathology score (SMI) was compared to changes in lung compliance in the bleomycin-treated segments. The overall pathology score showed a strong inverse correlation to both the compliance values measured at 7 weeks post-bleomycin (r_s_= −0.79, p=0.01, n=9 Fig. 9), in the corresponding segments and also in the percentage change of compliance from pre-treatment values at 7 weeks post-bleomycin (r_s_= −0.71, p=0.03, n=9) This correlation shows the close link between compliance and the pathology scores in this model.

### Pro-fibrotic environment prevails in bleomycin-treated lobes

**Fig. 10:**
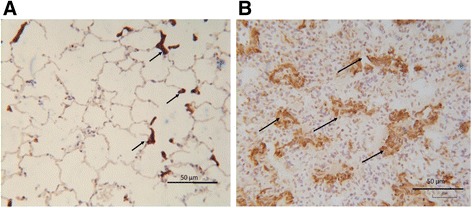
Myofibroblasts in bleomycin treated lung. Representative images of αSMA immunostaining seen in: saline-treated control lung segments (**a**), showing localization predominantly around the interstitial space of alveolar duct (arrowheads). In bleomycin-treated segments (**b**) αSMA positive cells were observed in remodelled fibrotic alveolar and formed organised bundles (arrows)

Myofibroblasts are considered to be a key cell type in driving the persistent and progressive fibrosis in IPF. The presence of contractile myofibroblasts was observed in damaged regions of the interstitial and alveolar space, based on intense positive staining for alpha smooth muscle actin (α-SMA) (Fig. 10b, arrowheads). Thin, spindle like projections of the positive α-SMA stain could also be observed in the remodelled alveolar regions. The staining pattern observed in the saline-treated control was as expected, with positive staining localising predominantly to around the interstitial space of the alveolar duct (Fig. 10a, arrowheads).

**Fig. 11:**
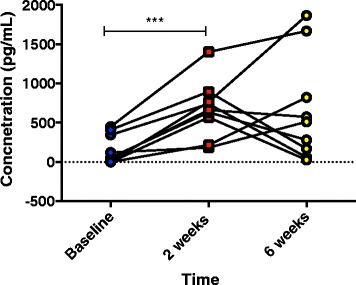
Changes to TGF- β signalling in bleomycin-treated segments. Levels of TGF- β protein were measured by ELISA using BAL samples from bleomycin-treated segments. The samples were taken at several key time points: baseline, or pre-bleomycin treatment, 2 weeks post-bleomycin and 6 weeks post-bleomycin. There was a significant increase in the amount of TGF- β at 2 weeks post bleomycin in all segments. At 6 weeks post injury, there was more variability in the level detected

The levels of the TGF-β protein were also measured in BAL samples taken at several key time-points from the bleomycin-treated lobes as an indicator of the level of fibrotic activity in the bleomycin-treated lobes (Fig. 11). As expected, TGF- β levels were significantly elevated from pre-treatment values at 2 weeks post injury (158 ± 62.7 vs. 672 ± 121 pg/mL, p=0.001, n=9, Fig. 11). At 6 weeks post-bleomycin, the response was much more varied: several sheep showed a further elevation in the levels detected at 2 weeks, some showed similar levels and other sheep showed a decrease back to baseline levels Fig. 11. Overall, the levels for all the bleomycin-treated segments at 6 weeks compared to baseline was increased, although this did not reach statistical (663 ± 226 vs. 158 ± 62.7 μg/mL, p=0.12, n=9, Fig. 11).

## Discussion

Despite recent advances in therapeutics available for IPF, there is a continued need to find more effective therapies to directly prevent or ideally reverse the fibrosis in the lungs. The development of novel therapies relies on how well experimental animal models are able to translate findings to clinically related outcomes but there is still much debate around the reliability of current animal models for pulmonary fibrosis. While functional assessments are key end-points in clinical trials and disease staging in IPF [24], only a few small animal models of pulmonary fibrosis incorporate long term lung functional measurements into their protocols. Here, we present a model that offers the ability to measure not only histological and biochemical changes in the lung, but also incorporates long-term lung function monitoring throughout the experiment. The segmental infusion approach used in the current model offers several key advantages over conventional small animal models, which typically induce fibrosis to the whole lung. Firstly, the model has the ability to temporally deliver infusions of bleomycin to discrete lung segments. This not only reduces the number of animals used for an experiment, but also improves statistical power by allowing for direct comparisons in the same animal between an internal normal lung control segment and a lung segment receiving an active agent. In addition, all stages of the disease, up to end-stage, can be examined in a small specific lung segment, while the untreated regions of the lung are healthy and can undertake normal respiratory functions of a healthy animal. This is an important consideration, as high mortality rates approaching 20-50 %, and significant initial weight loss are common issues experienced in murine bleomycin models which can confound injury responses in the lungs [11, 12, 25].

It is important to note that, as with any animal model, there are limitations to the sheep model that need to be considered. Foremost, in attempting to replicate a complex and poorly understood disease such as IPF, it is nearly impossible to fully recapitulate the exact the nature of the disease. Like other animal models of pulmonary fibrosis, the sheep model induces fibrosis within a relatively short time over a number of weeks, compared to the natural history of IPF where human patients take decades to develop the disease [1]. Animal models also largely fail to create the progressive nature of fibrosis seen in IPF where the pathology and lung function of IPF patients deteriorate relatively rapidly after initial diagnosis [4, 26]. Over time, the pulmonary fibrotic responses in animal models typically either plateau, or resolve, rather than progressively decline. Notwithstanding this, studies in animal models are still an important tool to help further our improved understanding of the disease and find suitable treatment and should be used synergistically to exploit the individual advantages each has to offer. Therefore, the distinguishing features described the sheep model should be used to complement other experimental systems in contributing to the understanding of pulmonary fibrosis.

The inclusion of physiological changes provides an additional, more clinically relevant end-point in our model to assess fibrotic changes throughout a study. Importantly, this is performed in a species with a respiratory system that more closely resembles the structure and function of the human lung [27, 28]. Lung function assessment in the current study shows that there was a significant drop in segmental lung compliance in the bleomycin treated segments, which persisted for seven weeks following bleomycin injury. Importantly, the lung function decline was significantly correlated with pathology scores at 7 weeks post bleomycin. Moreover, sheep that showed progressive declines in lung function over the study period were found to have the most severe pathology, as indicated by the SMI scoring data. This data is consistent with a number of early clinical studies, which demonstrate a correlation between structural and functional changes in IPF lungs [29, 30]. In these studies, lung function measurements of FVC, gas exchange (dLCO) and compliance were correlated to the extent of both fibrosis and cellularity in IPF patients [29, 30]. The association between deteriorating lung function and concomitant pathology has been ultimately linked with the survivability of patients [26, 31]. The overall significance of our findings was that sequential lung function assessment in this large animal model could serve as a reliable indication of the fibrotic changes occurring in the lung.

To our knowledge, there are no other large animal models for pulmonary fibrosis that examine histological and physiological changes in the lung. In small animal models, there are only a limited number of bleomycin models that investigate changes to lung function beyond the acute stages of injury. In the majority of previously published animal studies which utilize bleomycin as the fibrotic agent and include lung function measurements, bleomycin administration appears to have only had a minor impact on pulmonary mechanics and is generally only observable in the acute stage of injury, and not the later fibrotic stages [8, 9]. Indeed, very few bleomycin studies report physiological data as an end-point in pre-clinical investigations, which is an obvious limitation for these models. An exception is the recent study conducted in mice using a relatively high single dose of bleomycin which was able to detect measurable differences in lung function for up to 6 months after bleomycin administration [12]. In that study, while there were significant differences between control mice and bleomycin-treated mice in lung function indices of diffusion factor of carbon monoxide (DF_co_), total lung capacity, and compliance at six months after bleomycin administration, these parameters were all recovering towards normal at this time-point [12].

Interestingly, our lung function data is consistent with the TGF-β transgene model of pulmonary fibrosis in rats, which has been able to demonstrate a similar reduction in lung compliance for up to 7 weeks after the induction of fibrosis [32]. The degree of lung stiffness was also negatively correlated to pathology scores in the TGF-β rat model as we show for the sheep model [32]. This model utilizes adenovirus carrying active TGF-β1, which causes a transient overexpression of the protein to induce fibrosis [33]. This stimulates downstream pro-fibrotic responses, rather than continual TGF-β1 production, which result in the subsequent lung function decline. This also appears to be the situation in the current study, whereby transient TGF-β1 expression appeared to be sufficient to result in self-propagation of the fibrotic response that led to a loss of lung function. Whilst both models showed similar physiological changes, an asset of the sheep model we present here was that physiological data could be obtained without anesthesia or sedation and assessed throughout the entire experimental period, including the period just before cull. This allowed us to correlate compliance of individual lung segments with the fibrosis pathology in these segments.

TGF-β is an important pro-fibrotic cytokine which plays a central role in fibrosis [34]. The release of TGF-β from injured epithelial cells and other sources results in a fibrotic process with the accumulation of fibroblasts and myofibroblasts in the injured area. These cell types also express TGF-β to further stimulate this wound response [34, 35]. In the current study, we found that in TGF-β levels in BAL fluid were significantly increased in all the bleomycin treated lobes 2 weeks after bleomycin treatment. While at 6 weeks post treatment, the levels of TGF-β were much more variable, with some sheep showing increased expression, some unchanged, and others decreased expression. These levels did not correlate to other parameters, such as lung function, pathology or αSMA, expression (data not shown). As mentioned above, transient TGF-β1 expression appears to act as an important stimulus for the profibrotic response and can lead to the activation fibrogenic cytokines, including TNF-α, platelet-derived growth factor, and basic fibroblast growth factor [33]. We have previously shown by immunohistochemistry that TGF-β labelling is particularly strong in alveolar epithelial cells and hyperplastic Type II AEC cells, especially in regions of more severe fibrosis [13]. These cell types, as well as others in the lung, may contribute to the release of TGB- β into lung luminal fluids. Interestingly, there are differences in the literature regarding the duration of TGF-β expression in the BALF in bleomycin murine models. Early studies have shown peak expression at 14 days, returning to normal by 21 days after bleomycin [36], whilst others are found increasing levels up to this time point [37]. A more recent study suggests that peak levels are detectable early, i.e. 3 days post bleomycin, in the BAL and gradually recede [38]. This variability in responses may be indicative that active TGF-β eventually becomes bound to components of matrix such as the small proteoglycan biglycan [39], or fibronectin [40] and is active in these regions, and therefore is no longer detectable in the BAL fluid.

In this study, we found that overall injury and fibrotic pathology, as assessed by a modified Ashcroft’s scoring system, was similar in the bleomycin treated lobes at 7 weeks post injury to what was observed in our previous study at 2 weeks post bleomycin [13]. However, the inflammation scores detected at 7 weeks post-injury were significantly lower than that reported at 2 weeks post-injury [13]. The persistence of fibrosis at the 7 week time point was supported by data from both the hydroxyproline assay and Masson trichrome staining, which indicate an increase in collagen deposition at this time point compared with controls. The timing of the fibrotic and inflammation responses in the sheep bleomycin model is in accordance with the respective timeframes typically exhibited in bleomycin rodent models, where inflammation is observed up to 10–14 days post-bleomycin and then subsides to give rise to the fibrotic response, generally between day 21–28 after bleomycin [6, 10].

The finding that fibrosis persists and the overall cellularity of the lesions decreases over extended periods has been reported for bleomycin models in rodents that use both multi-hit [7, 11] and single dose methods [10, 38], but not by others using rodent models where the fibrosis resolved over 6 weeks post-injury [8, 9]. Whilst our findings are comparable with the studies showing persistent fibrosis [7, 10, 11, 38], one defining point of difference between the rodent and sheep models is the pattern of fibrosis observed in the sheep model. In general, intratracheal administration of bleomycin in rodent models typically produces a peribronchiolar pattern of fibrosis [8, 41], whilst IV administration of bleomycin to mice induces perivascular fibrosis [10, 41], of which both patterns are more commonly observed other lung diseases, such as asthma and COPD [23, 42]. In contrast, the patchy fibrosis observed in our study is found predominantly in the lung parenchyma, which is consistent with the pattern of fibrosis reported for IPF patients [1].

The sustained fibrotic response may reflect a prevailing profibrogenic environment within the bleomycin treated lung segments of sheep, as suggested by the increased positive αSMA immuno-staining found within the injured region of the segments. αSMA is a recognized indicator for the presence of myofibroblasts [43], a cell type which is known to be extensively localized in fibrotic lesions of IPF lungs [44]. It has been shown that the persistent presence of myofibroblasts appears to be a primary driver of the progression of fibrosis in IPF [45]. In bleomycin-induced fibrosis, the increased presence of the myofibroblast was found to occur in parallel to increased fibrotic injury [38]. The myofibroblast has been associated with the expression of several matrix proteins which contribute to matrix deposition [46].

Radiological examination of the lung is the primary method used to determine the presence of UIP and a diagnosis of IPF and is also used to help monitor the progression of fibrosis and stage of severity of disease [1]. Software programs, such as the one used in the current study allow for 3D reconstruction of the scanned area, which can then subsequently be measured and the specific density range can be quantified. In the current study, we chose to assess the radiological changes in the lungs of the sheep that had showed the worst lung function throughout the study to confirm that injury in the lung occurred in the segment treated with bleomycin. Additionally, this also enabled us to determine if this tool could be incorporated into our assessment of fibrosis, as has been successfully done in murine models [10, 32] The scan was performed ex-vivo for practical reasons, as it eliminated the need to anesthetize and transport the sheep for the procedure, similar to that previously published in a pre-clinical mice model [10]. In CT images, fibrotic lung tissue appeared denser compared to normal lungs, which was very clearly visualised on the images obtained from our sheep lung, showing a clear demarcation of the region locally treated with bleomycin compared to the remainder of the lung. This result suggested that ex-vivo CT scans could serve as a non-invasive tool for the assessment of fibrotic changes and intervention strategies in future studies, similar to that done by others [10, 32].

## Conclusion

In conclusion, the results from the current study demonstrated that a relatively sustained fibrotic response could be induced into isolated lung segments that also lead to a persistent, measurable change in lung compliance. Importantly, the changes in segmental compliance correlate strongly to pathology; therefore this parameter can serve as a reliable indicator of pathological changes in the lung. The assessment of lung function in this model is therefore likely to be a useful predictor of the efficacy of different intervention strategies against pulmonary fibrosis in future preclinical studies. The inclusion of more clinically relevant end-points into pre-clinical trials may aid in more accurate identification of potential drug candidates to translate into the clinic.

## Abbreviations

αSMA: alpha- Smooth muscle actin
BAL: bronchoalveolar lavage
BLM: bleomycin
Cseg: segmental compliance
ECM: extracellular matrix
HYP: hydroxyproline
IPF: idiopathic pulmonary fibrosis
LC: left caudal lobe
RC: right caudal lobe
SMI: semi-quantitative morphological index
TGF: Transforming growth factor
